# P-1791. Understanding Clinical Application of Whole Genome Sequencing for Antimicrobial Resistance

**DOI:** 10.1093/ofid/ofaf695.1960

**Published:** 2026-01-11

**Authors:** Samantha Giffen, Alison L Halpin

**Affiliations:** CDC, Atlanta, Georgia; Centers for Disease Control and Prevention, Atlanta, GA

## Abstract

**Background:**

Antimicrobial resistance (AR) remains a growing global concern, as detailed by U.S. Centers for Disease Control and Prevention (CDC) in the 2021-2022 “Antimicrobial Resistance Threats in the United States” report. Whole genome sequencing (WGS) is a powerful tool for enhancing our capacity to better address increasing threats of antimicrobial-resistant pathogens. However, integration of WGS and analysis into routine clinical microbiology laboratory testing is still evolving.

**Methods:**

We conducted a literature review to identify documented use cases of WGS data for patient management of antimicrobial-resistant bacterial healthcare-associated infections. The review was completed on PubMed using combinations of pre-defined key terms including “whole genome sequencing”, “antibiotic resistance”, “clinical management”, and “(Gram positive or Gram negative)”. The search was limited to articles published from 2020-2025 that covered a specific example of WGS data relating to understanding patient management.

**Results:**

Out of 27 total articles, 15 environmental and agricultural studies were excluded. These 12 articles covered Gram-positive and Gram-negative bacteria, causing various infections. All studies included a WGS component and some evaluation of AR, although the specific antibiotics evaluated varied. Few studies (n=3) evaluated a clinical outcome. Four papers discussed potential impact in patient management. However, this was not done in real-time. In most studies (n=10), WGS aided in detecting novel resistance patterns, enhancing our understanding of these mechanisms, and improving genotype-phenotype correlations for antibiotic susceptibility.

**Conclusion:**

These studies support that real-time WGS has the potential to be a powerful tool for clinical management in treating Gram-negative AR infections, providing rapid and precise genetic insights to guide antibiotic selection. There is an urgent need for development of standardized guidelines to allow labs to integrate WGS as a clinical test. Additionally, WGS contributes to protecting the public’s health by expanding our understanding of emerging resistance mechanisms and advancing the field’s ability to track and respond to AR threats.

**Disclosures:**

All Authors: No reported disclosures

